# Design of Fast Disintegrating Tablets of Prochlorperazine Maleate by Effervescence Method

**DOI:** 10.4103/0250-474X.57298

**Published:** 2009

**Authors:** S. B. Shirsand, Sarasija Suresh, M. S. Para, P. V. Swamy

**Affiliations:** Department of Pharmaceutical Technology, H. K. E. Society's College of Pharmacy, Sedam Road, Gulbarga-585 105, India; 1 Department of Pharmaceutics, Al-Ameen College of Pharmacy, Near Lalbagh Main Gate, Hosur Road, Bangalore-560 027, India

**Keywords:** Prochlorperazine maleate, fast disintegrating tablets, croscarmellose sodium, crospovidone

## Abstract

In the present work, fast disintegrating tablets of prochlorperazine maleate were designed with a view to enhance patient compliance by effervescent method. In this method, mixtures of sodium bicarbonate and anhydrous citric acid in different ratios along with crospovidone (2-10% w/w), croscarmellose sodium (2-10% w/w) were used as superdisintegrants. Estimation of prochlorperazine maleate in the prepared tablet formulations was carried out by extracting the drug with methanol and measuring the absorbance at 254.5 nm. The prepared formulations were further evaluated for hardness, friability, drug content uniformity and *in vitro* dispersion time. Based on *in vitro* dispersion time (approximately 13-21 s), two promising formulations (one from each super-disintegrant) were tested for *in vitro* drug release pattern in pH 6.8 phosphate buffer, short-term stability (at 40°/75% relative humidity for 3 mo) and drug-excipient interaction (IR spectroscopy). Among the two promising formulations, the formulation containing 10% w/w of crospovidone and mixture of 20% w/w sodium bicarbonate and 15% w/w of citric acid emerged as the overall best formulation (t_50%_ 6 min) based on drug release characteristics in pH 6.8 phosphate buffer compared to commercial conventional tablet formulation (t_50%_ 17.4 min). Short-term stability studies on the promising formulations indicated that there are no significant changes in drug content and *in vitro* dispersion time (p<0.05).

Many patients find it difficult to swallow tablets and hard gelatin capsules and thus do not comply with prescription, which results in high incidence of non-compliance and ineffective therapy. It is estimated that 70% of the population is affected by this problem[1]. Recent advances in novel drug delivery systems (NDDS) aimed to enhance safety and efficacy of drug molecule by formulating a convenient dosage form for administration and to achieve better patient compliance. One such approach is fast dissolving tablets (FDT)[1–4]. Prochlorperazine maleate (PCZM) is a phenothiazine antipsychotic and widely used in prevention and treatment of nausea, vomiting including that associated with migraine or drug-induced emesis[5]. The concept of formulating fast dissolving tablets containing prochlorperazine maleate offers a suitable and practical approach in serving desired objective of faster disintegration and dissolution characteristics with increased bioavailability. Upon ingestion, the saliva serves to rapidly disperse/dissolve the dosage form. The saliva containing the dissolved/dispersed medicament is then swallowed and the drug is absorbed in the normal way. Some drugs are absorbed from mouth, pharynx and esophagus as the saliva passes down into the stomach[6]. In such cases, bioavailability is significantly greater than those observed from conventional dosage form.

PCZM was a gift sample from Mehta Pharmaceuticals, Mumbai, India. Croscarmellose sodium (CCS) and crospovidone (CP) were gift samples from Wockhardt Research Centre, Aurangabad, India. Directly compressible mannitol (Pearlitol SD 200), microcrystalline cellulose (MCC) and sodium stearyl fumarate (SSF) were generous gifts from Strides Acrolabs, Bangalore, India. All the other chemicals used were of analytical reagent grade.

For the preparation of fast disintegrating tablets by effervescent method[7], all the ingredients (except SSF and purified talc) were accurately weighed and sifted through # 44 mesh separately. Sodium bicarbonate and anhydrous citric acid were pre-heated at a temperature of 80° to remove absorbed/ residual moisture and were thoroughly mixed in a mortar to get a uniform powder and then added to other ingredients. The ingredients after sifting through sieve No. 44 were thoroughly mixed in a tumbling cylindrical blender (fabricated in our laboratory). The blend thus obtained was directly compressed into tablets of 150 mg weight (at ambient temperature and humidity conditions) on a 10-station rotary machine (Clit, Ahmedabad, India) using 8 mm round flat punches. The tablets were prepared according to the formulae shown in Table 1.

**TABLE 1:** COMPOSITION OF FAST DISINTEGRATING TABLETS OF PROCHLORPERAZINE MALEATE

Ingredients (mg/ tablet)	Formulation Code*								
	
	EC_0_	ECP_1_	ECP_2_	ECP_3_	ECP_4_	ECCS_1_	ECCS_2_	ECCS_3_	ECCS_4_
Prochlorperazine Maleate	5.0	5.0	5.0	5.0	5.0	5.0	5.0	5.0	5.0
Sodium bicarbonate	22.5	22.5	15.0	22.5	30.0	22.5	15.0	22.5	30.0
Citric acid	15.0	15.0	7.5	15.0	22.5	15.0	7.5	15.0	22.5
Crospovidone	--	--	3.0	9.0	15.0	--	--	--	--
Crosarmellose sodium	--	--	--	--	--	--	3.0	9.0	15.0
Microcrystalline cellulose	--	30.0	30.0	30.0	30.0	30.0	30.0	30.0	30.0
Aspartame	1.5	1.5	1.5	1.5	1.5	1.5	1.5	1.5	1.5
Talc	3.0	3.0	3.0	3.0	3.0	3.0	3.0	3.0	3.0
Sodium stearyl fumarate	1.5	1.5	1.5	1.5	1.5	1.5	1.5	1.5	1.5
Pineapple flavor	1.5	1.5	1.5	1.5	1.5	1.5	1.5	1.5	1.5
Pearlitol SD-200	100.0	70.0	82.0	61.0	40.0	70.0	82.0	61.0	40.0

* A batch of 60 tablets was prepared for each formulation

Twenty tablets were selected at random and weighed individually. The individual weights were compared with the average weight for determination of weight variation[8]. Hardness and friability of the tablets were determined by using Monsanto hardness tester and Roche friabilator, respectively. For content uniformity test, ten tablets were weighed and powdered. The powder equivalent to 5 mg of PCZM was extracted into methanol and liquid was filtered (Whatmann No. 1 filter paper). The PCZM content in the filtrate was determined by measuring the absorbance at 254.5 nm after appropriate dilution with methanol. The drug content was determined using the standard calibration curve. The mean percent drug content was calculated as an average of three determinations[9]. For determination of *in vitro* dispersion time, one tablet was placed in a beaker containing 10 ml of pH 6.8 phosphate buffer at 37±0.5° and the time required for complete dispersion was determined[10]. IR spectra of PCZM and its formulations were obtained by KBr pellet method using Perkin-Elmer FTIR series (Model 1615) spectrophotometer in order to rule out drug-carrier interactions.

*In vitro* dissolution of PCZM fast disintegrating tablets was studied in USP XXIII type-II dissolution apparatus (Electrolab, Model-TDT 06N) employing a paddle stirrer at 50 rpm using 900 ml of pH 6.8 phosphate buffer at 37±0.5° as dissolution medium[11]. One tablet was used in each test. Aliquots of dissolution medium (5 ml) were withdrawn at specified intervals of time and analyzed for drug content by measuring the absorbance at 255.5 nm. The volume withdrawn at each time interval was replaced with fresh quantity of dissolution medium. Cumulative percent of PCZM released was calculated and plotted against time. The studies were performed in triplicate.

Dissolution efficiency is widely used parameter for evaluation of *in vitro* dissolution data[12]. It is defined as the area under the dissolution versus time curve up to a certain time ‘t’ expressed as a percentage of area of the rectangle described by 100% dissolution in the same time. DE_10min_ values were calculated from the dissolution profiles of promising and conventional commercial formulation. Dissolution efficiency is expressed as the Eqn., $\text{DE}=\int_{0}^{\text{t}}\frac{yd}{100t}x100$.

Short-term stability studies on the promising formulations (ECP_4_ and ECCS_4_) were carried out by storing the tablets in an amber coloured glass vial with rubber stopper at 40°/75% RH over a 3 mo period. At an interval of 1 mo, the tablets were visually examined for any physical changes, changes in drug content and *in vitro* dispersion time. The data was subjected to statistical analysis by using student's ‘t’ test to find out any significant changes in the above parameters (p<0.05).

Fast disintegrating tablets of PCZM were prepared by effervescent method employing CP and CCS as super-disintegrants along with mixtures of sodium bicarbonate and anhydrous citric acid in different ratios with MCC. Directly compressible mannitol (Pearlitol SD 200) was used as a diluent to enhance mouth feel. A total of eight formulations and a control formulation EC_0_ (without super-disintegrant) were designed. As the blends were free flowing (angle of repose <30°, and Carr's index <15%) tablets obtained were of uniform weight (due to uniform die fill), with acceptable variation as per IP specifications i.e., below 7.5%. Drug content was found to be in the range of 95 to 102%, which is within acceptable limits. Hardness of the tablets was found to be 2.6 to 3.0 kg/cm^2^. Friability values below 1% were an indication of good mechanical resistance of the tablets. Among all the designed formulations, two formulations, viz., ECP_4_ and ECCS_4_ were found to be promising and displayed an *in vitro* dispersion time ranging from 13 to 21 s, which facilitates their faster dispersion in the mouth.

Overall, the formulation ECP_4_ containing 10% w/w of CP along with mixture of sodium bicarbonate 20% w/w and anhydrous citric acid 15% w/w and 20% w/w of microcrystalline cellulose was found to be promising and has shown an *in vitro* dispersion time of 13 s when compared to control formulation (EC_0_) which shows 118 s for *in vitro* dispersion (Table 2).

**TABLE 2:** EVALUATION OF FAST DISINTEGRATING TABLETS

Formulation Code	Parameters					
	
	Hardness* (kg/cm^2^)	Thickness (mm)	Friability (%)	*In vitro* dispersion time* (Sec)	Percent drug content* (%)	Weight variation
EC_0_	2.8±0.20	2.15	0.64	117.87±2.48	97.77±0.62	(146 - 150 mg) within the IP limits of ±7.5%
ECP_1_	2.8±0.264	2.17	0.70	98.69±1.60	97.76±0.73
ECP_2_	2.83±0.152	2.36	0.55	59.79±1.63	99.03±0.78
ECP_3_	2.92±0.05	2.37	0.42	44.66±2.37	97.96±1.38
ECP_4_	2.63±0.25	2.41	0.49	13.29±1.02	95.68±0.59
ECCS_1_	2.83±0.20	2.34	0.60	104.97±2.40	101.14±1.30
ECCS_2_	2.64±0.305	2.39	0.64	62.42±0.90	101.27±0.74
ECCS_3_	2.76±0.305	2.73	0.54	51.47±0.66	99.42±1.02
ECCS_4_	2.64±0.208	2.40	0.50	20.42±1.25	99.46±0.71

* Average of three determinations. Formulations ECP_4_ and ECCS_4_ were selected as the best formulations and used for further studies.

*In vitro* dissolution studies on the promising formulations (ECP_4_ and ECCS_4_), the control (EC_0_) and CCF (Stemitil 5 mg, Nicolas Piramal Health Care, Baddi, HP, India) were carried out in pH 6.8 phosphate buffer, and the various dissolution parameter values viz., percent drug dissolved in 5, 10 and 15 min (D_5_, D_10_ and D_15_), dissolution efficiency at 10 min (DE_10min_)[12], t_50%_, t_70%_ and t_90%_ are shown in Table 3 and the dissolution profiles depicted in fig. 1. This data reveals that overall, the formulation ECP_4_ containing 10% w/w of CP along with effervescent mixture has shown nearly three-fold faster drug release (t_50%_ 6 min) when compared to the CCF of prochlorperazine maleate (t_50%_ 17.4 min) and released five-times more drug than the control formulation in 10 min.

**Fig. 1:**
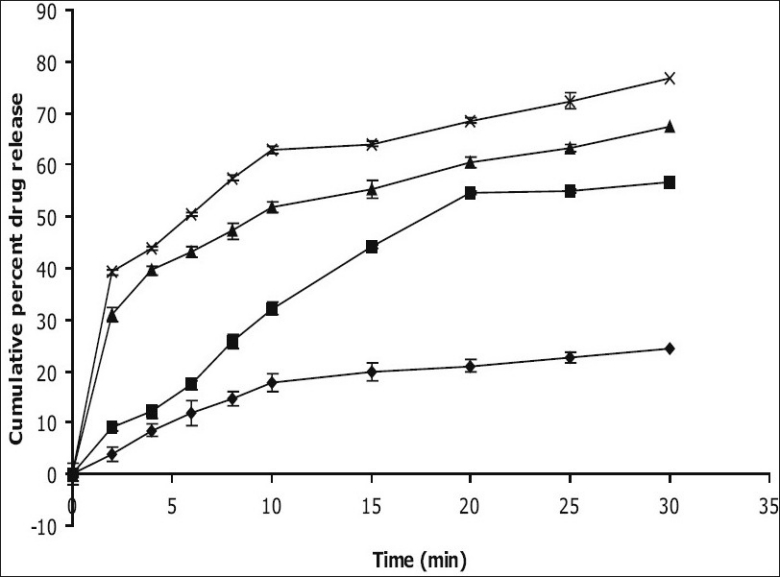
*In vitro* cumulative percent drug release versus time profile of promising formulations Plot showing percent cumulative release of promising prochlorperazine maleate formulations in pH 6.8 phosphate buffer. EC_0_ (-♦-), CCF (-▪-), ECCS_4_ (-▲-), ECP_4_ (-χ-). The values expressed are average of three readings.

**TABLE 3:** *IN VITRO* DISSOLUTION PARAMETERS IN PH 6.8 PHOSPHATE BUFFER

Formulation code	Dissolution Parameters						
	
	D_5_ (%)	D_10_ (%)	D_15_ (%)	DE_10min_ (%)	t_50%_ (min)	t_70%_ (min)	t_90%_ (min)
EC_0_	10.00	18.00	20.00	26.28	>30	>30	>30
ECP_4_	47.00	63.00	64.00	34.71	6.00	22.00	>30
ECCS_4_	42.00	54.00	59.00	33.38	8.20	26.00	>30
CCF	24.00	32.00	44.00	24.75	17.40	>30	>30

EC_0_=control formulation, CCF=conventional commercial formulation, D_5_= percent drug released in 5 min, D_10_=percent drug released in 10 min, D_15_= percent drug released in 15 min, DE_10_min= dissolution efficiency in 10 min, t_50%_= time for 50% drug dissolution, t_70%_= time for 70% drug dissolution.

IR spectroscopic studies indicated that the drug is compatible with all the excipients. The IR spectrum of pure drug shows the characteristic absorption bands around 3299 cm^−1^ for hydroxyl group of acid, 1735 and 1663 cm^−1^ for carbonyl group of acid, while the aromatic and aliphatic CH stretching were observed around 2970, 2953, 2938 and 2917 respectively. The IR spectrum of ECP_4_ showed all the characteristic peaks of PCZM pure drug, thus confirming that no interaction of drug occurred with the components of the formulation. Short-term stability studies of the above formulations indicated that there are no significant changes in drug content and *in vitro* dispersion time at the end of 3 mo period (p<0.05).

Further *in vivo* studies in healthy human volunteers are being planned in order to ascertain the enhanced bioavailability of the drug PCZM from the designed promising fast disintegrating tablet formulation (ECP_4_).
